# Detection of *Pseudomonas aeruginosa* in sputum headspace through volatile organic compound analysis

**DOI:** 10.1186/1465-9921-13-87

**Published:** 2012-10-02

**Authors:** Pieter C Goeminne, Thomas Vandendriessche, Johan Van Eldere, Bart M Nicolai, Maarten LATM Hertog, Lieven J Dupont

**Affiliations:** 1 Department of Lung Disease, UZ Leuven, Leuven, Belgium; 2 Biosyst-MeBios, University of Leuven, Leuven, Belgium; 3 Department of Microbiology, UZ Leuven, Leuven, Belgium; 4 Pulmonary Medicine, University Hospital Gasthuisberg, Herestraat 49, Leuven, B-3000, Belgium

**Keywords:** Bronchiectasis, Chronic colonization, Gas chromatography mass spectrometry, Cystic fibrosis, Non-cystic fibrosis

## Abstract

**Introduction:**

Chronic pulmonary infection is the hallmark of Cystic Fibrosis lung disease. Searching for faster and easier screening may lead to faster diagnosis and treatment of *Pseudomonas aeruginosa (P. aeruginosa)*. Our aim was to analyze and build a model to predict the presence of *P. aeruginosa* in sputa.

**Methods:**

Sputa from 28 bronchiectatic patients were used for bacterial culturing and analysis of volatile compounds by gas chromatography–mass spectrometry. Data analysis and model building were done by Partial Least Squares Regression Discriminant analysis (PLS-DA). Two analysis were performed: one comparing *P. aeruginosa* positive with negative cultures at study visit (PA model) and one comparing chronic colonization according to the Leeds criteria with *P. aeruginosa* negative patients (PACC model).

**Results:**

The PA model prediction of *P. aeruginosa* presence was rather poor, with a high number of false positives and false negatives. On the other hand, the PACC model was stable and explained chronic *P. aeruginosa* presence for 95% with 4 PLS-DA factors, with a sensitivity of 100%, a positive predictive value of 86% and a negative predictive value of 100%.

**Conclusion:**

Our study shows the potential for building a prediction model for the presence of chronic *P. aeruginosa* based on volatiles from sputum.

## Introduction

Chronic pulmonary infection is the hallmark of Cystic Fibrosis (CF) lung disease. Preventing or treating chronic infection plays a key role in these patients. Previous studies showed that *Pseudomonas aeruginosa* (*P. aeruginosa)* infection is associated with lower forced expiratory volume in one second (FEV_1_) during childhood, faster decline in FEV_1_ during childhood and reduced survival [1-9]. Chronic *P. aeruginosa* infection is normally preceded by an intermittent presence of the bacteria [10]. Early eradication during this period is important to delay chronic colonization [11]. To accomplish early eradication, regular surveillance cultures of sputum are indicated. For non-expectorating patients, oropharyngeal swabs or bronchoalveolar lavage can be used [10].

One of the difficulties measuring successful eradication is proving that the bacteria are completely eliminated from the patient, rather than just temporarily suppressed to a low level that is not detectable, particularly by cough swab [12,13]. Sputum culture can be false negative due to overgrowth of other bacteria or (maintenance) treatment with inhaled or oral antibiotics [14,15]. A positive culture should not be regarded as a gold standard for diagnosing (chronic) *P. aeruginosa* infection in CF patients with bronchiectasis and repeated culturing is still a cornerstone of a possible classification based on both bacterial cultures and specific antibody analysis [16]. Repeated culturing is also the cornerstone in non-CF bronchiectasis for the diagnosis of chronic *P. aerugiosa* although different definitions are used [17]. Therefore, other techniques aiming at diagnosis and follow-up of bacterial infection are being investigated. One approach is detection of volatile organic compounds (VOCs) produced by bacteria. *P. aeruginosa* may be detected by analyzing VOCs produced *in vitro* (Table 1), although the many studies addressing this question measured a variable range of VOCs. Breath or sputum samples are more challenging to investigate as many factors might influence the VOCs spectrum (eg. recent meal, other bacteria, concomitant medication). A few studies investigating *in vivo* samples (breath, sinus mucus and sputum) (Table 1) suggest that *P. aeruginosa* can be detected via the breath using not only hydrogen cyanide as a single marker [18], but also other biomarkers [19,20]. These *in vivo* studies use bacterial cultures as a gold standard to assess *P. aeruginosa* presence in the sample, not taking into account chronically colonized patients with a false negative sputum culture.

**Table 1:** **Literature overview of volatile organic compounds found in *in vitro *and *in vivo *studies in samples with *Pseudomonas aeruginosa***

***In vitro***	**Volatiles**	**reference**
	acetaldehyde, acetic acid, acetone, ammonia, ethanol, dihydrogen sulfide, dimethyl disulfide, dimethyl sulfide, methyl mercaptan	[21]
	ammonia, hydrogen cyanide, methyl mercaptan	[22]
	hydrogen cyanide	[23]
	2-aminoacetophenone, ammonia, ethanol, formaldehyde, hydrogen sulfide, isoprene, methyl mercaptan, trimethylamine	[24]
	2-aminoacetophenone, 2-pentanone, 4-methylphenol, acetic acid, acetone, acetonitrile, ethanol, ethylene glycol, indole	[25]
	1-butanol, 1-undecene, 2-butanone, 2-heptanone, 2-nonanone, 2-undecanone, 3-methyl-1-butanol, toluene	[26]
	2-aminoacetophenone	[27]
	1-undecene, 2-aminoacetophenone, 2-butanone, 2-nonanone, 2-undecanone, 3-methyl-1-butanol, 4-methyl-quinazoline, butanol, dimethyl disulfide, dimethyl trisulfide, methyl mercaptan, toluene	[28]
	2-propanol	[29]
	2-aminoacetophenone, dimethyl disulfide, dimethylpyrazine, dimethyl sulfide, undecene	[30]
	Methyl thiocyanate	[20]
*In vivo*		
breath	hydrogen cyanide	[18]
breath	Methyl thiocyanate	[20]
breath	2-propanol	[29]
sinus mucus	2-aminoacetophenone, 2-methylbutyric acid, 3-hydroxy-2-butanone, acetamide, acetic acid, acetone, dimethyl disulfide, dimethyl sulfide, dimethylsulfone, hydrogen sulfide, indole, isovaleric, phenol, propanoic acid	[30]
sputa	1-heptene, 2-nonanone, 2,4-dimethyl-heptene, 3-methyl-1-butanol, limonene	[31]

**Figure 1:**
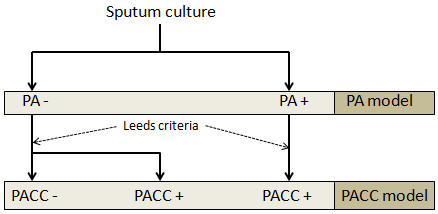
**Volatile analysis flow-chart.** Sputum culture was first analyzed for the presence of *P. aeruginosa*. The PA model analyzed positive versus negative *P. aeruginosa* patients. In a second step the Leeds criteria were applied to each patient to determine *P. aeruginosa* chronic colonization [32]. The PACC model compared chronically colonized patients with non-chronically colonized patients. PA = *P. aeruginosa*; PACC = *P. aeruginosa* chronic colonization.

The aims of our study were to predict sputum culture positivity for *P. aeruginosa* in patients with bronchiectasis (PA model) and to predict chronic colonization status with *P. aeruginosa* in patients with bronchiectasis (PACC model) by analysis of the presence of VOCs (Figure 1).

## Materials and methods

### Patients

Consecutive patients who were visiting the outpatient clinic with CF and non-CF bronchiectasis were included in the study. They were asked to collect their morning sputa, after rinsing their mouth with water and before breakfast, and to bring it to the outpatient clinic. A part of the sputum was used for routine bacterial culture. The second part was used for VOCs analysis within two hours of outpatient clinic visit. Clinical records were reviewed to assess chronic colonization status according to the Leeds criteria [32]. In brief, chronic colonization is diagnosed when more than 50% of the months, when samples had been taken, were *P. aeruginosa* culture positive. Informed consent was obtained from all patients. Approval was obtained from the local ethical committee of UZ Leuven, Belgium (B51060 - B32220084152).

### Detection of volatiles

From every patient 20 grams of morning sputum was transferred into a 10 mL glass headspace vial (Filter Service, Belgium) within 4 hours from collection, flushed with nitrogen gas and sealed using crimp-top caps with TFE/silicone septa seals (Filter Service, Belgium). Prior to solid phase micro extraction (SPME), the sputum samples were incubated for 24h at 37°C in a heated tray oven. Headspace volatiles were extracted by exposing a divinylbenzene-carboxen-polydimethylsiloxane SPME fiber (DVB-CAR-PDMS, 50/30 μm film thickness; Supelco Inc., Bellefonte, PA, USA) to the vial headspace for 60 min at 37°C. The headspace in our samples is defined by the gaseous constituents of the closed space above the sputum. Every 100 measurements, a new fiber was used. Each fiber was conditioned according to manufacturer’s description.

The determination of the VOCs was performed on an Agilent 6890N gas chromatograph (GC) (Agilent Technologies, Santa Clara, USA) coupled to an Agilent 5973 Network Mass Selective Detector (MS) (Agilent Technologies, Santa Clara, USA). Automated headspace SPME extraction was performed with an MPS-2 robotic arm (MPS2, Gerstel Multipurpose Sampler, Mülheim an der Ruhr, Germany). After extraction, the VOCs were thermally desorbed into a split/splitless injector heated at 250°C and equipped with a SPME liner (0.75 i.d., Supelco Inc., USA). To detect low concentration volatiles, splitless injection was used. Splitless injection was performed for 0.5 min at 75 mL/min and the fiber was further exposed in the injector for 5 min for thermal conditioning.

Separation was done on an Optima-5-MS capillary column (30 m x 0.25 mm i.d. x 0.25 μm d_f_) (Macherey-Nagel, Germany). Helium was used as carrier gas under a constant flow of 1.0 mL/min. The GC temperature program started isothermal at 35°C for 3 min and was then ramped to 250°C at a rate of 10°C min^-1^. Finally, the temperature was kept isothermal at 250°C for 5 min. The total run time was 29.50 min. The GC interface temperature was 280°C.

Mass spectra in the 15 to 350 m/z range were recorded at a scanning speed of 4.15 scans cycles per second. The MS source and quadrupole temperatures were 230°C and 150°C respectively. The chromatography and spectral data were evaluated using the MSD ChemStation Software (Agilent Technologies, Santa Clara, USA) and AMDIS v. 2.1 (Automated Mass Spectral Deconvolution and Identification System, NIST, Gaithersburg, MD, USA). Only those compounds with a signal to noise ratio > 20 and that could be identified through comparison with the spectral library NIST having a match and reversed match percentage > 80% and from which additionally the spectrum was manually controlled, were included in the analysis. The volatile compounds were identified by comparing the experimental spectra with those of the National Institute for Standards and Technology (NIST98 v. 2.0, Gaithersburg, MD, USA) and by retention indices. The retention time is the characteristic time it takes for a specific volatile to pass through the system. The (Kovats) retention index of a compound is its retention time normalized to the retention times of adjacently eluting n-alkanes. They help to identify components by comparing experimentally found retention indices with known values. The Kovats retention index is used to allow other analytical laboratories to compare measured values. We evaluated VOCs with a molecular weight higher than 30. Lower molecular weight VOCs (such as Hydrogen Cyanide) could not be evaluated as too many small compounds were co-eluting in the beginning of the chromatogram. Therefore, it was not possible to determine their presence in a reliable way (even with deconvolution programs). Hydrocarbon standards (C_8_ to C_20_ in hexane, Sigma-Aldrich, Steinheim, Germany) were injected using the same GC-MS method to determine the retention indices of the individual compounds using a modified Kovats method [33].

### Bacterial culturing

Sputa were inoculated on standard culture media (Blood agar with optochin disc, Mannitol Salt agar and MacConkey agar). Selective culture media were used for *Haemophilus* spp. (*Haemophilus* agar) *Burkholderia cepacia* complex (Mast *B. cepacia* complex agar) and fungi (Sabouraud agar)

### *Pseudomonas aeruginosa* (PA) model

For the PA model, we compared patients with a *P. aeruginosa* positive sputum culture at study to those with a negative *P. aeruginosa* sputum culture at study visit (Figure 1).

### *Pseudomonas aeruginosa* chronically colonized (PACC) model

For the PACC model, we compared patients with a known *P. aeruginosa* colonization according to the Leeds criteria to those without *P. aeruginosa* colonization at study visit (Figure 1) [32].

### Multivariate data analysis

All data was evaluated using multivariate data analysis techniques, including Principal Component Analysis (PCA) and Partial least-squares discriminant analysis (PLS-DA). The former is an unsupervised explorative method which is based on the principle of latent variables. It transforms large multivariate datasets of correlated variables into a new (reduced) dataset containing orthogonal (uncorrelated) variables only, named principal components. The latter is then used to reveal the relation of the samples to a given parameter, where the predictor variable is used in the calculation of the latent variables. The goal is to describe as much of the response variation and to search for directions that are relevant with respect to the predictor variable. The obtained PLS model can be further used to predict the predictor variable response for unknown samples. Data preprocessing steps included mean centering and weighing of all variables by their standard deviation to give them equal variance. In order to evaluate every dataset before analysis, a PCA was conducted to detect possible outlying samples by means of the 95% Hotelling’s T^2^ limit. Hotelling's T-squared statistic is a generalization of Student’s t statistics that is used in multivariate hypothesis testing. Two samples were discarded from the dataset due to technical failure during measurement. PLS-DA, a supervised technique, was used to discriminate between non-infected patients versus patients infected with *P. aeruginosa* or chronically colonized patients versus noncolonized patients*.* In order to test the performance of the models, a segmented (4 x 7) cross-validation was applied. The quality of the model was evaluated by using the R^2^-value between measured and predicted. The Variable Identification (VID) coefficients were calculated to identify possible biomarkers. The VID coefficient was calculated as the correlation coefficient between each original X-variable and the Y-variable as predicted by the PLS-DA model [34]. The VID is therefore important to understand what the potential relevance of each aroma compound is with respect to the predictor variable. PCA and PLS-DA analyses were performed using Unscrambler vs 9.8 (CAMO Technologies Inc., Woodbridge, USA).

## Results

### Population

During the study period 30 patients were recruited and sputum was analyzed of 28 patients (male (43%); average age 29 y ± 12; 11% non-CF bronchiectasis and 89% CF). Two samples were discarded from the dataset due to technical failure during measurement. Bacterial culturing of the 28 patients showed that 14 patients had *P. aeruginosa* in their sputa (50%) collected at the time of the study. Five patients did not grow *P. aeruginosa* in sputum culture but were known to be chronically colonized according to the Leeds criteria [32]. The remaining nine patients had no history of having *P. aeruginosa* cultured in their sputum. The patients with chronic *P. aeruginosa* colonization had an average IgG for *P. aeruginosa* of 40 AU.

All but one patient were taking antibiotics as treatment, either with a single or a combined scheme of antibiotics (68% on chronic macrolide therapy, 54% on inhaled tobramycin and/or on inhaled colistimethate; 11% on oral penicillines; 14% on oral quinolones; 7% on oral cefalosporins, 4% on oral clindamycin and 7% on oral co-trimoxazoles.) Two of the patients on oral antibiotics took their oral antibiotic treatment as maintenance therapy and the other nine received it due to an exacerbation they had suffered. In addition to *P. aeruginosa*, bacterial culture isolated *Staphylococcus aureus* in 36%*, Aspergillus fumigatus* in 29%, *Achromobacter xylosoxidans* in 11%, *Haemophilus influenza* in 7% and *B. cepacia* complex in 7%.

## GC-MS results

Around one hundred aroma compounds were detected using the deconvolution software AMDIS. This resulted in 61 VOCs (Table 2) of which the retention indexes (RI) were also checked.

**Table 2:** Overview of all volatile organic compounds studied with their respective retention time (RT), Kovats retention index (RI) and variable identification coefficients (VID) in the PACC model

**Name**	**RT**	**KRI**	**VID**
1R-α-pinene	8,872	937,3333	0.42
2,2,6-trimethyl-octane	9,363	963,52	0.42
dodecane	13,29	1200	0.40
terpinen-4-ol	13,03	1183,14	0.40
1-undecene	11,6	1091,77	0.37
3,7-dimethyl-1,6-octadien-3-ol	11,73	1099,704	0.32
2,6,7-trimethyl- decane	11,03	1058,378	0.31
indole	14,69	1296,944	0.31
toluene	5,377	759,4782	0.31
ethanol	1,746	< 600	0.31
3-hydroxy-2-butanone	4,261	697,5298	0.30
acetic acid	2,673	609,3811	0.28
amylene hydrate	3,046	630,086	0.27
caryophyllene	16,55	1438,148	0.26
1-methyl-4-(1-methylethyl)-cyclohexanol	12,95	1178,121	0.26
2,5-dimethyl-2,5-hexanediol	8,466	915,68	0.25
2-nonanone	11,56	1089,816	0.25
acetone	1,859	< 600	0.22
2-ethyl-1-hexanol	10,54	1029,248	0.22
2-heptanone	7,947	889,1041	0.21
2-ethoxy-2-methyl-propane	2,801	616,4863	0.21
phenylethyl alcohol	11,97	1115,187	0.18
1-octen-3-ol	9,66	979,36	0.18
4-methyl octane	7,46	865,5206	0.17
1-butanol, 3-methyl-, acetate	7,686	876,4649	0.15
1-butanol, 3-methyl-	4,796	727,2273	0.14
d-limonene	10,6	1032,445	0.14
Eucalyptol	10,64	1034,991	0.14
6-methyl-2-heptanone	9,192	954,4	0.14
Thymol	14,62	1292,222	0.12
Benzeneacetaldehyde	10,76	1042,214	0.12
2-hexanone	5,859	786,2337	0.11
2,4-dimethyl-1-heptene	7,004	843,4383	0.11
5-methyl-2-(1-methylethyl)-cyclohexanone	12,63	1157,529	0.10
2,4-dimethyl-heptane	6,599	823,8257	0.09
Pyrollidine	3,583	659,8945	0.08
2,3-dimethyl-heptane	7,3	857,7724	0.06
2,6-dimethyl-7-octen-2-ol	11,27	1072,114	0.05
3-methyl-2-pentanone	5,085	743,2695	−0.03
2-undecanone	14,61	1291,597	−0.04
3-octanone	9,737	983,4667	−0.08
methyl isobutyl ketone	4,806	727,7824	−0.08
phenol	9,733	983,2533	−0.12
3-methyl-3-buten-1-ol	4,686	721,1213	−0.14
2-pentyl-furan	9,89	991,6267	−0.15
3-methyl butanal	3,194	638,3014	−0.16
1-propanol	2,213	< 600	−0.19
3-methyl-, (ethyl ester) butanoic acid	7,213	853,5593	−0.20
octane	6,122	800,7264	−0.22
1-butanol	3,487	654,5656	−0.22
2-butanone	2,492	< 600	−0.23
2-pentanone	3,776	670,6078	−0.24
thiocyanic acid, methyl ester	4,188	693,4777	−0.24
2-methyl-,(ethyl ester) butanoic acid	7,151	850,5569	−0.26
2-methyl butanal	3,355	647,2384	−0.27
ethyl acetate	2,709	611,3794	−0.28
hexane	2,528	601,3322	−0.38
dimethyl tetrasulfide	13,5	1214,583	−0.43
dimethyl disulfide	4,878	731,7791	−0.46
dimethyl trisulfide	9,5	970,8267	−0.47
2-methyl-pentane	2,259	< 600	−0.59

Volatile Organic Compounds (VOCs) were ranked according their VID with high values indicating a positive correlation with Pseudomonas aeruginosa infection and negative values indicating a negative correlation; KRI = Kovats Retention Index.

### Multivariate data analysis

#### PA model

**Figure 2:**
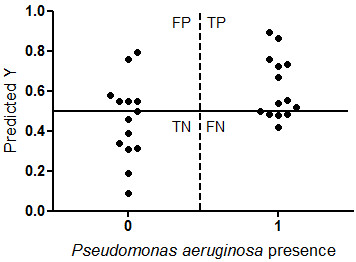
**PA model.** Y-axis shows prediction of *P. aeruginosa* presence of our model based on VOC analysis. X-axis shows presence of *P. aeruginosa* based on sputum culture. Sensitivity was 72%, Specificity was 40%, positive predicted value was 63% and negative predicted value was 67%.

In the PA model, *P. aeruginosa* positivity was based on sputum culture positivity for *P. aeruginosa* at study visit, excluding the patients known to be chronically colonized from the *P. aeruginosa* positives. The PA model showed an explained variance of 95% after 9 PLS-DA Factors but showed an unstable validation. It also showed less good prediction for the presence of PA in sputum culture with high number of false positives and false negatives. Sensitivity was 72%, specificity was 40%, positive predicted value was 63% and negative predicted value was 67% (Figure 2).

#### PACC model

**Figure 3:**
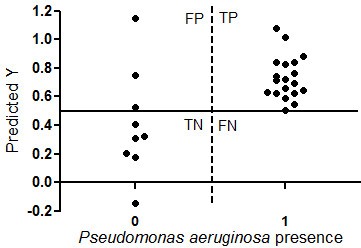
**PACC model.** Y-axis shows prediction of chronic colonization with *P. aeruginosa* of our model. X-axis shows chronic colonization status with *P. aeruginosa* based on sputum Leeds criteria. Model predicts with a sensitivity of 100%, specificity of 67%, positive predicted value of 86% and negative predicted value of 100%. FN = False negatives; FP = False positives; TN = True negatives; TP = True positives.

Our PACC model included all *P. aeruginosa* chronically colonized patients, even if sputum culture at study visit was negative. The PACC model can explain the colonization status with *P. aeruginosa* with an explained variance of 95% with 4 PLS-DA Factors, and a stable validation. It showed a good prediction of presence with *P. aeruginosa*. The PACC model had no false negatives, but there were three false positive (Figure 3). This means our PACC model has a sensitivity of 100%, a specificity of 67%, a positive predictive value of 86% and a negative predictive value of 100%.

### Volatile analysis of the PACC model

**Figure 4:**
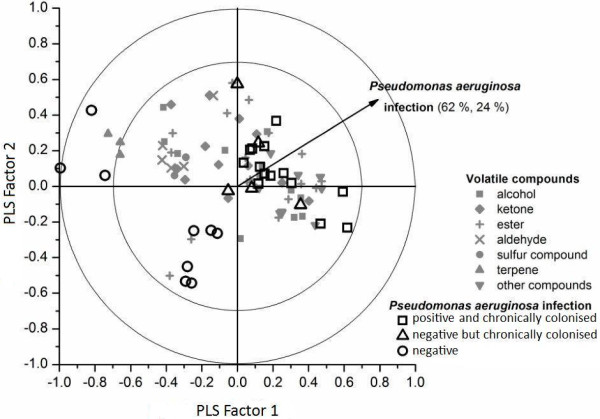
**Biplot using the first two PLS-DA factors.** The plot shows a good separation of *P. aeruginosa* positive chronic colonized patients (triangles and squares) and *P. aeruginosa* negative patients (circles). Significant correlation of volatiles is suggested when volatiles project between r=0.75 (inner circle) and r=1 (outer circle). The vector shows the direction where volatiles are positively correlated with chronic *P. aeruginosa*. The pattern of volatiles could explain *P. aeruginosa* infection in 86% using the first two PLS-DA factors (62% and 24%). X and Y axis both show partial least square regression r. Each PLS factor explains 10% and 6% of the X-variation respectively. The light gray symbols visualize the volatile organic compounds, sorted by structure. Squares: Chronic colonization and positive sputum cultures for *P. aeruginosa* at the time of study. Triangles: Chronic colonization but negative sputum culture for *P. aeruginosa* at the time of study. Circles: Negative for *P. aeruginosa.*

Based on the PLS-DA, the Variable Identification (VID) coefficients were calculated in order to examine the relationship between each VOC and the presence of *P. aeruginosa.* VID coefficients showed a positive and negative correlation with the presence of certain VOCs, although most correlation loadings were low (Table 2). This can also be perceived in the correlation loadings plots (Figure 4). Using two principle compounds, 86% of *P. aeruginosa* presence can be explained through the PACC model. There’s a clear separation between *P. aeruginosa* positive and negative patients in the correlation loadings plot (Figure 4). VOCs analysis shows that the five largest negative correlations can be seen for the sulphur compounds dimethyl disulfide (VID = −0.46), dimethyl trisulfide (VID = −0.47) and dimethyl tetrasulfide (VID = −0.43) and two other compounds: hexane (VID = −0.38) and 2-methyl pentane (VID = −0.59). The five largest positive correlations were found for the terpenes 1-undecene (VID = 0.37) and 1-α-pinene (VID = 0.42) and the compounds dodecane (VID = 0.40), terpinen-4-ol (VID = 0.40) and 2,2,6-trimethyl-octane (VID = 0.42)(Table 2).

Exclusion of the non-CF bronchiectatic patients from the PLS analysis, analyzing only the CF population did not change the results in terms of positions of the VOCs and amount of X (=VOCs) and Y (=*P. aeruginosa*) variation explained (data not shown).

## Discussion

Our study shows that it may be possible to use the presence of VOCs in sputum to assess the presence of *P. aeruginosa* and colonization status with *P. aeruginosa*. Analysis showed that not a single but a pattern of VOCs are linked to the presence of *P. aeruginosa*. VOCs that were positively associated with *P. aeruginosa* included the terpenes 1-undecene, 1-α-pinene, dodecane, terpinen-4-ol and 2,2,6-trimethyl-octane. A more pronounced negative correlation can be seen for the sulphur compounds dimethyl disulfide, dimethyl trisulfide and dimethyl tetrasulfide with the addition of hexane and 2-methyl-pentane. The results of the PACC model showed a sensitivity and negative predictive value of 100%. This suggests that, based on VOCs analysis, our model is able to predict chronic colonization with *P. aeruginosa*. Some of the patients known with chronic colonization of *P. aeruginosa* had a negative sputum culture for *P. aeruginosa* at study visit. This suggests that gas chromatography – mass spectrometry may be more sensitive than bacterial culturing.

Previous studies have shown that several VOCs in sputa, breath and mucus may indicate the presence of *P. aeruginosa *[18,29-31]*.* Our study results confirm that most of these VOCs were present in sputum from patients with *P. aeruginosa*, but none of these VOCs were highly specific for the presence of *P. aeruginosa*. We could not identify one single VOC that was representative for the presence of *P. aeruginosa* presence.

In our study, the presence and absence of a library of 61 VOCs was identified and found to discriminate between patients with and without *P. aeruginosa* in sputum. Some of the VOCs we identified in the sputum headspace samples were the same as those found in other studies. If we compare the results with the study of Savelev et al. we can find their suggested markers in our samples [31]. They looked for specific biomarkers, showing the highest individual sensitivity for 2-nonanone. Although our specific aim was to look for a prediction model, rather than searching and evaluating individual candidate biomarkers, we found a similar positive correlation with 2-nonanone (VID= 0.25), limonene (VID= 0.14), 2,4-dimethyl-heptene (VID=0.11) and 3-methyl-1-butanol (VID= 0.14).

A clear distinction needs to be made between VOCs analysis of bacterial cultures (*in vitro* studies) and patient *in vivo* sample analysis. One typical example is 2-aminoacetophenone. 2-aminoacetophenone is known for its sweet grape-like odour. On culture plates growing *P. aeruginosa *[27,28], its odour increases when adding tryptophan. This is because 2-aminoacetophenone is an intermediate in the biosynthetic pathway for quinazolines, a pathway branching from the tryptophan catabolic pathway. Conversely, only one *in vivo* study could show its presence in trace quantities [30]. This indicates that the VOCs profile produced by *P. aeruginosa in vivo* may differ from its *in vitro* VOCs production and cannot be extrapolated from *in vitro* to *in vivo* analysis purposes, as culture media can have an impact on VOCs. Moreover, most *in vitro* studies are explorative studies, describing the spectrum of VOCs in different bacterial cultures without assessing them as biomarkers (such as dimethyl disulfide and dimethyl sulfide), with the exception of hydrogen cyanide [21,23], 2-propanol [29] and methyl thiocyanate [20]. Hydrogen cyanide, 2-propanol and methyl thiocyanate were also found in *in vivo* samples (breath). Hydrogen cyanide was not evaluated as our GC-MS results only allowed reliable evaluation of VOCs with a molecular weight higher than 30. For 2-propanol, the isomer 1-propanol could be detected but was also seen in samples without *P. aeruginosa*. Methyl thiocyanate (or thiocyanic acid, methyl ester) was not associated with *P. aeruginosa* in our samples. Shestivska et al. could not find methyl thiocyanate in some *P. aeruginosa* strains. This means that methyl thiocyanate is strain specific and might explain its absence in our study population.

The different results on the presence of VOCs shown in some previous studies (Table 1) raises the question if not a single VOC is indicative of *P. aeruginosa* presence but rather a pattern of VOCs, as suggested by our results. However we did not analyze VOCs with a molecular weight lower than 30. Recently, strong evidence showed that hydrogen cyanide could be used as a biomarker, showing significant higher *in vivo* concentrations in most strains of *P. aeruginosa *[18]. This biomarker could then be used in the detection of *P. aeruginosa* in breath*,* whether or not in combination with CH_3_SCN (methyl thiocyanate) as possible biomarker [20]. Further research is warranted to identify a single biomarker or a pattern of VOCs (“a breathogram”). This would mean the addition of a new tool for the diagnosis of (chronic) *P. aeruginosa* infection and the monitoring of response to treatment (eg eradication therapy) [35].

The use of novel devices using the breath end portion of a normal spirometry measurement to perform a chromatographic preseparation, followed by an ion mobility spectrometry (IMS) or devices allowing fast analysis of breath using a selected ion flow tube mass spectrometry (SIFT-MS) make it fast and feasible to do VOCs analysis [36,37]. SIFT-MS has the advantage of being fast and having high sensitivity. It can also determine the end-tidal breath phase by quantification of water vapour in breath samples while the soft ionization technique allows easy analysis of high moisture samples such as breath. IMS has the disadvantage of not knowing what chemical compound is seen unless a large database with standards is available, but it has been proven that IMS is also fast and can show a fingerprint, characteristic for an infection [38].

A limitation of our study might be the impact other variables have on VOCs such as antibiotic therapy and other bacteria. Bacterial culture results from all our patients showed a great diversity and variability without a distinct pattern of bacterial co-existence between patients. More importantly, our statistical design, using PLS-DS, minimizes the impact of variables such as antibiotic therapy and other bacteria. PLS-DS reveals the relation of the samples to a given parameter, particularly *P. aeruginosa*.

Our findings of terpenes and terpenoids in sputum headspace are interesting as they are common constituents of food. Alpha-pinene for example is detected in fruits and pepper. Although we asked the patients to produce their sputa after rinsing their mouth and before breakfast, we cannot reliably say this was done by the patient. However, if the detected VOCs would indeed be related to food, this would mean that all patients with *P. aeruginosa* had the same food VOCS constituents in their breath.

Quantification of the VOCs was also not performed. To perform quantification for complex matrices, the use of internal standards or standard additions is recommended. Using only a few internal standards, representing the main chemical classes and extrapolating the results to all volatiles in the sample, can cause serious errors. Ideally SPME quantification would require us to focus on a few volatiles (which was not our aim) and use isotopically labeled analogues as standards. Although we did not quantify, all samples were processed and analyzed in a same manner, reducing the variability due to the methods. This results in a variability mainly due to the sample itself.

Another important issue that should be taken into consideration is that sputum might be contaminated by saliva, influencing the results of the VOC analysis. This has been proven for breath analysis, where important contamination of alveolar breath exhaled via the mouth can occur [39]. Wang et al. showed that both mouth- and nose-exhaled breath analyses are needed to identify the major source of a certain VOC. We tried to minimize the effect of saliva contamination by asking the patient to rinse their mouth prior to sputum production. Nonetheless, finding a biomarker for *P. aeruginosa* in mouth VOCs would still be interesting as current literature suggests that a migration from *P. aeruginosa* is seen from the upper to the lower airways prior to colonization [40].

## Conclusion

We showed that building a model for the prediction of *P. aeruginosa* presence is possible and might even identify known chronic colonized patients as *P. aeruginosa* where sputum culture cannot show its presence. Based on literature overview and our results, we believe that not the presence of a single VOC is indicative of *P. aeruginosa* presence but rather a pattern of VOCs. Follow-up of patients, producing a “breathogram” might be a promising future perspective, but needs further research, using new devices such as spirometry combined with chromatographic preseparation and subsequent ion mobility spectrometry.

## Abbreviations

CF: Cystic fibrosis; FEV_1_: Forced expiratory volume in one second; GC-MS: Gas chromatography – mass spectrometry; IMS: Ion mobility spectrometry; *P. aeruginosa*: *Pseudomonas aeruginosa*; PACC: *Pseudomonas aeruginosa* chronic colonization; PCA: Principal component analysis; PLS-DA: Partial least square discriminant analysis; RI: Retention index; RT: Retention time; SIFT-MS: Selected ion flow tube mass spectrometry; SPME: Solid phase micro extraction; VID: Variable identification; VOCs: Volatile organic compounds.

## Competing interest

None of the authors has a financial relationship with a commercial entity that has an interest in the subject of the presented manuscript.

## Authors’ contribution

PG performed the acquisition and analysis of the data, designed the study and wrote the manuscript. TV aided in the data acquisition and data processing, performed part of the analysis and reviewed the article. JVE was involved in the design of the study and reviewed the article. MH contributed importantly to the interpretation of the data and critically revised the manuscript. BN was involved in the design of the study and critical revision of the manuscript. LD was involved in the design and critical revision prior to submission. All authors read and approved the final manuscript.
